# Amyloid β oligomers elicit mitochondrial transport defects and fragmentation in a time-dependent and pathway-specific manner

**DOI:** 10.1186/s13041-016-0261-z

**Published:** 2016-08-17

**Authors:** Yanfang Rui, James Q. Zheng

**Affiliations:** 1 Department of Cell Biology, Emory University School of Medicine, 615 Michael Street, Atlanta, GA 30322 USA; 2 Department of Neurology, Emory University School of Medicine, Atlanta, GA 30322 USA; 3 Center for Neurodegenerative Diseases, Emory University School of Medicine, Atlanta, GA 30322 USA

**Keywords:** Alzheimer’s disease, Transport, Fragmentation, Hippocampus, HDAC6, Drp-1, GSK3β

## Abstract

Small oligomeric forms of amyloid-β (Aβ) are believed to be the culprit for declined brain functions in AD in part through their impairment of neuronal trafficking and synaptic functions. However, the precise cellular actions of Aβ oligomers and underlying mechanisms in neurons remain to be fully defined. Previous studies have identified mitochondria as a major target of Aβ toxicity contributing to early cognitive decline and memory loss in neurodegenerative diseases including Alzheimer’s disease (AD). In this study, we report that Aβ oligomers acutely elicit distinct effects on the transport and integrity of mitochondria. We found that acute exposure of hippocampal neurons to Aβ oligomers from either synthetic peptides or AD brain homogenates selectively impaired fast transport of mitochondria without affecting the movement of late endosomes and lysosomes. Extended exposure of hipoocampal neurons to Aβ oligomers was found to result in mitochondrial fragmentation. While both mitochondrial effects induced by Aβ oligomers can be abolished by the inhibition of GSK3β, they appear to be independent from each other. Aβ oligomers impaired mitochondrial transport through HDAC6 activation whereas the fragmentation involved the GTPase Drp-1. These results show that Aβ oligomers can acutely disrupt mitochondrial transport and integrity in a time-dependent and pathway-specific manner. These findings thus provide new insights into Aβ-induced mitochondrial defects that may contribute to neuronal dysfunction and AD pathogenesis.

**Electronic supplementary material:**

The online version of this article (doi:10.1186/s13041-016-0261-z) contains supplementary material, which is available to authorized users.

## Introduction

Alzheimer’s disease (AD) is a progressive neurodegenerative brain disorder that is characterized by two hallmarks: intracellular neurofibrillary tangles and extracellular amyloid-β (Aβ) plaques in the regions of the brain that are responsible for learning and memory. Aβ, peptides of 39–43 amino acids produced by the sequential cleavage of β- and r-secretase at the C terminal of amyloid precursor protein, are believed to play a central role in the neurodegeneration and subsequent cognitive abnormalities of AD brains [1]. Recent evidence indicates that small oligomeric species of Aβ are responsible for impaired brain functions in the early stages of AD, in part through their detrimental actions on neuronal trafficking and synaptic functions [2–5]. Increasing evidence suggests that mitochondrial abnormalities play a critical role in AD pathogenesis. However, our understanding of various Aβ effects on mitochondria and their contribution to AD brain's dysfunction and disease progression remains incomplete.

Mitochondria are a cell’s vital organelle of energy production and they participate in diverse cellular functions ranging from Ca^2+^ homeostasis to cell apoptosis. Mitochondria are mainly produced in the soma and transported to the specific subcellular regions via microtubule (MT)-based fast transport [6, 7]. In neurons, mitochondria are seen accumulated at the pre- and post-synaptic sites where they function in synaptic transmission and plasticity [8–12]. Such subcellular localization of mitochondria involves spatiotemporal regulation of MT-based motors as well as interactions with other cytoskeletal structures such as the actin cytoskeleton and intermediate filaments [6]. The half-life of mitochondria is about 1 month and mitochondrial fusion and fission provide a mechanism for mitochondrial renewal and degradation [10, 13, 14]. Therefore, timely and proper delivery of new mitochondria from the cell body is important to support the health and function of mitochondria in neurons.

Disruption of mitochondrial transport, structure, and function is believed to be a major contributor to many neurodegenerative diseases [10, 15]. Interestingly, disrupted axonal transport has been observed prior to the major pathological hallmarks such as senile plaques in AD [16, 17]. In many of these cases, axons exhibit swellings and varicosities with accumulated mitochondria and other organelles. On the other hand, damaged mitochondria dynamics in dendrite has been associated with the synaptic deficit in AD [18, 19]. Mitochondria that are stuck in the swellings/varicosities often look unhealthy and appear to undergo degradation and fragmentation. The cellular mechanisms that lead to defected mitochondrial transport and structure remain to be fully defined. In this study, we utilized cultured hippocampal neurons to investigate the acute effects of small Aβ oligomers on mitochondria. In particular, we examined the effects of Aβ oligomers prepared from synthetic peptides and the human AD brain homogenates on mitochondrial transport and morphology. Our data show that Aβ oligomers generated two distinct effects on mitochondria that are temporally segregated: transport impairment preceded the fragmentation. We further investigated the underlying mechanisms that mediate the specific effects of small Aβ oligomers on mitochondria.

## Methods

### Hippocampal culture, transfection and live imaging

All the experiments involving the use of vertebrate animals were carried out in accordance to the NIH guideline for animal use and have been approved by the Institutional Animal Care and Use Committee of Emory University. Hippocampal neurons from embryonic day 18 rats were obtained as described previously [20]. About ~200,000 cells were plated in a 35 mm glass bottom culture dish (Warner Instruments, Hamden, CT) that were coated with 100 μg/ml poly-D-lysine (Sigma, St. Louis, MO). Hippocampal neurons were cultured in the Neurobasal medium containing B27 and Glutamax (Invitrogen) and typically imaged after 3–7 days in culture. Transfection of hippocampal neurons was performed using the CalPhos Mammalian Transfection Kit (Clontech, Mountain View, CA), which was typically done one day before the imaging [19]. The DNA constructs for transfection were prepared by plasmid maxi kit (Qiagen, Valencia, CA). Mito-DsRed and Mito-GFP were provided by Dr. Zheng Li at NIH/NIMH. All the labeling and imaging were carried out in Krebs’–Ringer’s buffer (KRB, in mM: 150 NaCl, 5 KCl, 2 CaCl_2_, 1 MgCl_2_, 10 glucose, and 10 HEPES, pH 7.4) [19, 21, 22]. For simultaneous imaging of mitochondria and lysosome trafficking, neurons were sequentially labeled by MitoTracker Green (20 nM for 20 min; Invitrogen, Eugene, OR) and LysoTracker Red (100 nM for 30 min; Invitrogen), followed by three rinses and additional 10 min incubation at 37 °C before imaging.

Dual-channel fluorescent time-lapse recordings were performed on an inverted microscope (TE2000, Nikon) equipped with a multispectral imager (Dual-View; Optical Insights) using either a 40× with 1.3 numeric aperture (NA) S Fluor oil immersion objective or a 60× 1.4 NA Plan Apo oil immersion objective with identical settings between the control and experimental groups. Time-lapse images were captured with a charge-coupled device (CCD) camera (SensiCam QE, Cooke Scientific) using the IPLab imaging software (BD Biosciences). We typically recorded neurons at a sampling rate of one frame every 5 s for 5 min, with the CCD exposure at 50 ms and 2x2 binning. For each experiment, a population of neurons was imaged for a 5 min control period before the application of Aβ molecules, followed by another 5 min time-lapse recording after Aβ exposure for 30 min or 2 h. All of the experiments were performed on the microscope stage with the 35 mm dish housed in a temperature controlled chamber (Warner Instruments, New Haven, CT) with the temperature set at ~35 °C. For different inhibitors, we applied the inhibitor 20 min before the onset of Aβ exposure. Tubacin was provided by Dr. Stuart Schreiber at Broad Institute of Harvard and MIT through the support by the Initiative for Chemical Genetics, National Cancer Institute. SB415286 and SB216763 were purchased from Tocris Bioscience (Ellisville, MO), Trichostatin A (TSA) was from Sigma (St. Louis, MO), and MS-275 was from Selleckchem (Houston, TX).

We quantified the Aβ effects on organelle movement by determining the percentage of moving mitochondria before and after Aβ exposure [19, 22]. Here, quantification was done by repeatedly playing back the time-lapse sequences and counting the number of moving mitochondria or other organelles (moved >3 μm) in each 5 min time-lapse sequence. We normalized the number of moving mitochondria or other organelles in the 5-min sequence against that before the Aβ application. A value of 100 % indicates that same numbers of moving mitochondria or vesicles were observed in both recording periods [19, 22]. To generate the movement traces of mitochondria and other organelles, ImageJ (National Institutes of Health, Bethesda, MD) was used to first process the image sequence using the Zprojection function (maximum intensity), followed by division against the first frame to produce the final image of the moving traces [22]. The movement traces were also used to confirm the number of moving mitochondria over the 5 min time-lapse. Kymograph analysis was done using ImageJ with the multiple kymograph plugin written by J. Rietdorf (FMI Basel) and A. Seitz (EMBL Heidelberg). Here, Zprojection of the time-lapse sequence was used to identify the moving organelles and segmented line regions of interest (ROIs) were created from the movement traces. These segmented line ROIs were then applied to the time-lapse sequence to produce kymographs that allow the measurement of the average speed of each moving organelle.

### Aβ oligomeric preparation and treatment

Aβ oligomers were prepared from either synthetic Aβ_1-42_ (American Peptide Company Inc, Sunnydale,CA) or recombinant Aβ_1-42_ (r-Peptide Company, Bogart, GA) according to the procedure described previously [23]. In brief, Aβ_1-42_ was dissolved in hexafluoro-2-propanol (HFIP) and aliquoted to microcentrifuge tubes. HFIP was subsequently removed by evaporation in a speed-vacuum and desiccated Aβ aliquots were stored at -20 ^o^C. To make Aβ oligomers, Aβ_1-42_ was dissolved in DMSO to make a 5 mM stock solution. The stock solution was then diluted to 100 μM with KRB and kept at 4 ^o^C for 24 h before use. Bath application of Aβ was achieved through a two-step dilution procedure. First, the Aβ stock solution was diluted in KRB to twice the designated concentration (2× working stock). The 2× working stock solution was warmed to ~35 ^o^C and then gently added to and mixed with the bath saline of the cells in an equal volume to reach the desired final concentration. In a typical experiment, 1 ml of the 2× stock solution was added to 1 ml of the bath solution in the culture/imaging dish on the microscope stage.

Postmortem human brain tissues were obtained from the tissue bank of the Emory Alzheimer’s Disease Research Center and the Emory Center for Neurodegenerative Disease. Frozen tissues from the frontal cortex of age- and gender-matched control (*n* = 3) and AD (*n* = 3) subjects were provided. Control subjects had no clinical history of neurological disease and were free of neurodegenerative disease pathology at autopsy. AD subjects met both CERAD [24] and NIA-Reagan [25] criteria for the neuropathologic diagnosis of AD. The frozen tissues were weighted and homogenized in ice-cold phosphate-buffered saline at 20 g/100 ml using a Dounce homogenizer (A size pestle for 15 strokes, B size pestle for 15 strokes). Homogenate was then sonicated 3 times for 5 s (power around 4), vortexed, and centrifuged for 5 min at 3000 g. The supernatant was collected, aliquoted in eppendorf tubes, and stored at -80 ^o^C. Before application to the cells, each aliquot was thawed and centrifuged at 10,000 rpm for 5 min to remove any aggregates and precipitates. Similar to the application of Aβ oligomers from synthetic peptides, we applied the soluble human brain homogenate through a two-step dilution procedure: the 2× working stock and then 1:1 (volume:volume) to the cells.

### Silver staining, western blotting and immunoprecipitation

Aβ samples (200 ng each) from American Peptide Company or r-Peptide Company were loaded to sample buffer with 50 mM DTT and heated at 85 °C for 5 min. Samples were loaded and fractioned by SDS-PAGE on 10–20 % Tris-Tricine gel (Invitrogen) for 90 min. After electrophoresis, the gel was briefly rinsed with ultrapure water and subjected to silver staining using SilverXpress® Silver Staining Kit (Invitrogen). For western blotting, different Aβ samples from synthetic peptides or human brain tissues were loaded in equal volume and fractioned by SDS-PAGE using 10–20 % Tris-Tricine gel and subsequently transferred to nitrocellulose membrane. The membrane was boiled for 10 min in PBS and blocked with 5 % non-fat dry milk in TBS with 0.05 % Tween-20 for 1 h at room temperature. The membrane was then incubated with a mixture of two anti-Aβ antibodies: 6E10 (1:1000) and 4G8 (1:1000) antibodies (Signet, Dedham, MA) in blocking buffer overnight at 4 °C. Bound antibodies were detected by HRP-conjugated secondary antibody, visualized by chemiluminescence using ECL (Thermo Scientific, Rockford, IL), and quantified using the gel analysis routine of ImageJ software (NIH).

To deplete Aβ molecules from the soluble human brain homogenate, we performed immunoprecipitation (IP) using Protein A-agarose beads (Santa Cruz). Here, the homogenate samples were incubated with 10 μl (2 μg) polyclonal anti-Aβ antibody A8326 (Sigma) for 1 h at 4 °C, followed by incubation with Protein A-agarose beads at 4 °C overnight on a rocker platform. The samples were subjected to centrifugation at 2500 rpm for 5 min at 4 °C to allow the collection of the supernatant (referred to as Thru 1st) and pellet (referred to as Output 1st) respectively. Parts of the supernatant from the first round IP (Thru 1st) was used to test the effects on organelle trafficking. Another round of IP was done with the remaining supernatant to obtain the second round IP supernatant (Thru 2st) and pellet (Output 2st). All these samples after IP were examined by western blot as described above.

## Results

### Aβ oligomers rapidly impair mitochondrial transport

**Fig. 1:**
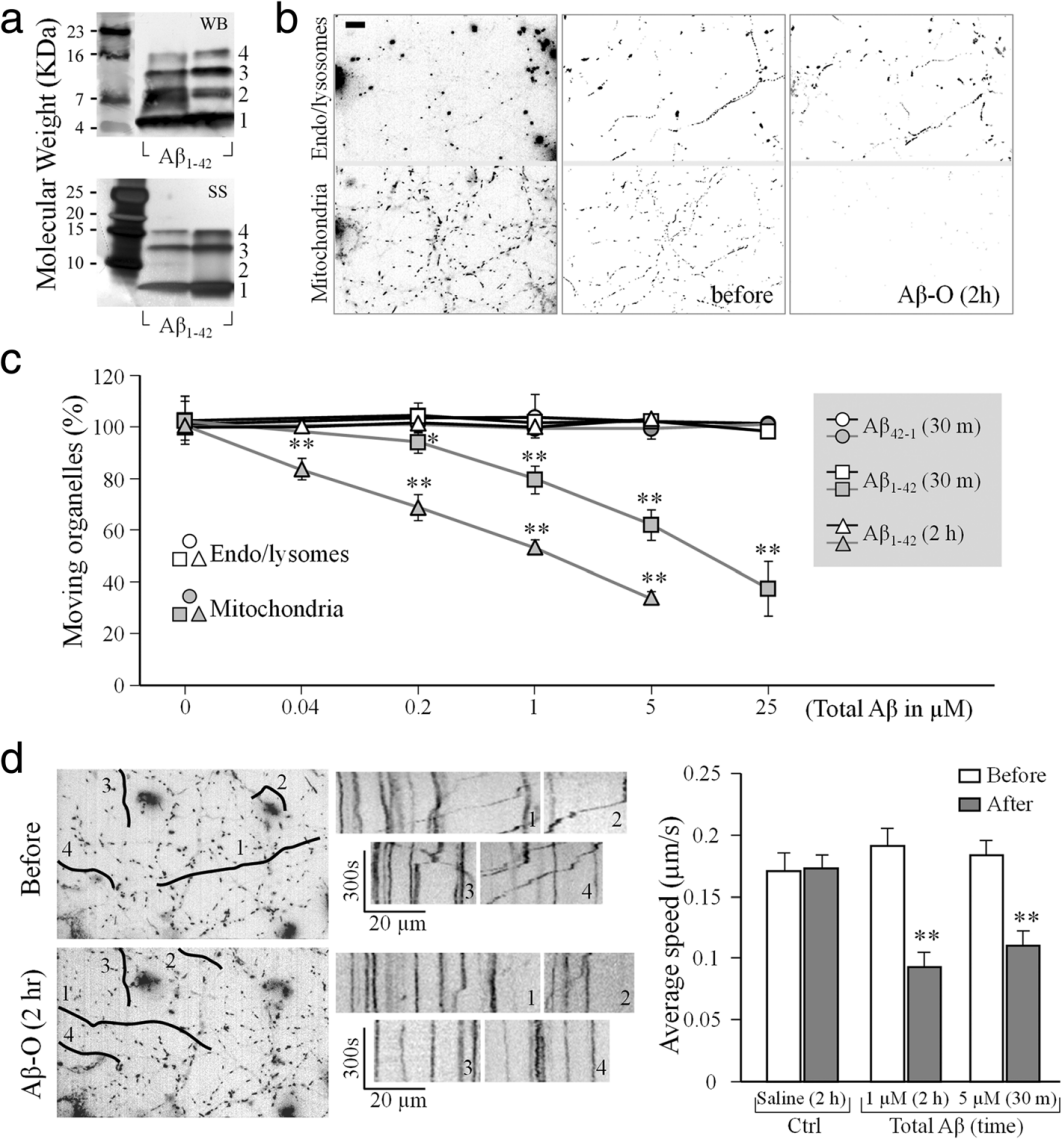
Selective inhibition of fast transport of mitochondria by Aβ oligomers. **a** Western blotting (WB, upper panel) and silver staining (SS, lower panel) showing the presence of Aβ monomers (1) and small Aβ oligomers, including dimmers (2), trimmers (3), and tetramers (4) in our Aβ oligomeric preparation (Aβ–O) from synthetic Aβ_1-42_. **b** Representative images showing hippocampal neurons with their late endosomes/lysosomes (endo/lysosomes; upper panels) and mitochondria (lower panels) labeled by LysoTracker and MitoTracker (left panels), as well as their movement tracings generated from 5 min time-lapse sequences before (middle panels) and after (right panels) 2 h exposure to 1 μM Aβ–O. Scale bar: 10 μm. **c** Quantitative analysis showing the dose- and exposure time-dependent Aβ–O effects on the fast transport of mitochondria and endo/lysosomes. Aβ_42-1_ was the negative control. All the data were normalized to the number of moving organelles in the 5-min time-lapse sequence before different treatments (see Methods). Each condition was repeated at least three times from different rounds of cultures and was averaged from 10 -20 cells. Error bars indicate the standard deviation (SD). *p < 0.05 and **p < 0.005 in comparison to the corresponding control (Student’s *t-*test). **d** Representative images (left) and kymographs (middle) showing the movement of mitochondria before and after Aβ oligomers 1 μM 2 h treatment. The kymographs are produced using the segmented lines labeled on the left images. Averaged transport speeds of mitochondria after different treatments are shown in the bar graph on the right. Data are from three dishes containing around total 60–150 moving mitochondria. Error bars indicate the standard error of the mean (SEM). **p < 0.005 when compared to the respective control period (Student’s *t-*test)

We double labeled mitochondria and late endosomes/lysosomes of cultured hippocampal neurons with MitoTracker and LysoTracker, and examined the effects of Aβ oligomers on the movement of these organelles [19, 22]. We first tested Aβ oligomers prepared from synthetic Aβ_1-42_ and both western blotting and silver staining confirmed the presence of small Aβ oligomers, including dimers, trimers, and tetramers, in this Aβ oligomeric preparation from synthetic Aβ_1-42_ (hereafter referred to as Aβ-O; Fig. 1a). Aβ-O also contained a large portion of monomers that exert no effect on mitochondrial transport as we showed before [19]. We estimated from different batches of Aβ-O that the small oligomers account for ~10 % of the total Aβ concentration [19]. We found that exposure of cultured hippocampal neurons to Aβ-O (1 μM total Aβ, ~100 nM of oligomers) markedly inhibited the fast transport of mitochondria without affecting late endosomes/lysosomes (hereafter referred to as endo/lysosomes) of the same neurons, as evidenced by the movement tracings from the 5-min time-lapse sequences (Fig. 1b). To quantitatively determine the effects of Aβ-O on the transport of these two populations of organelles, we utilized a simplified method in which the percentage of moving organelles was quantified from the 5-min time-lapse sequence (see Methods). We found that moving endo/lysosomes accounted 50.2 % ± 1.3 % (Mean ± SD) and 51.4 % ± 1.9 % (Mean ± SD) of the total endo/lysosomes before and after 2 h exposure to Aβ-O (1 μM total Aβ), respectively. However, the number of moving mitochondria dropped from 24.3 % ± 2.7 % (Mean ± SD) to 14.4 % ± 0.8 % (Mean ± SD) of the total mitochondria after 2 h exposure to Aβ-O.

To better depict the effects of Aβ-O on organelle transport, we normalized the percentage of moving organelles after different treatments to that before the treatments. As shown in Fig. 1c, the inhibitory effect of Aβ-O on mitochondrial transport was rapid (e.g., 30 min) and dose-dependent. Significant impairment of mitochondrial transport was observed at 0.04 and 1 μM total Aβ concentrations (corresponding to 4 nM and 100 nM of Aβ oligomers) for 2 h and 30 min exposure, respectively (*p* < 0.005 comparing to that before Aβ-O application, Student’s *t*-test). Higher doses of Aβ-O were found to cause severe impairment of mitochondrial transport, whereas the transport of endo/lysosomes labeled by LysoTracker remained unaffected. Importantly, no effect was observed for the reverse peptide Aβ_42-1_ prepared the same way. Consistent with previous findings [22, 26], the impairment of mitochondrial transport by Aβ-O was abolished by inhibition of GSK3β using 1 mM LiCl (Additional file 1: Figure S1).

In addition to reducing the number of moving mitochondria, Aβ-O also slowed down the moving mitochondria. Kymograph analysis showed that the average speed of moving mitochondria was reduced to about half of that of the control period (Fig. 1d). It should be noted that all of our experiments were performed on relatively high density hippocampal cultures that contain mixed populations of highly elaborated and branched axons and dendrites. As a result, it is nearly impossible to distinguish between the MT plus end-directed anterograde and minus end-directed retrograde movements in these processes. The speed reported here represents the average of mitochondrial transport in both directions and from both axonal and dendritic processes. Consistently, Aβ-O did not significantly affect the speed of endo/lysosomes movement. We found that the average speed of endo/lysosomes was 0.20 ± 0.16 μm/s (Mean ± SD) before and 0.21 ± 0.20 (Mean ± SD) μm/s after Aβ–O treatment. Together, these results indicate that transport impairment of Aβ-O on mitochondria was not a result of non-specific and gross disruption of the transport machinery, and may involve specific signaling pathways that target mitochondria.

**Fig. 2:**
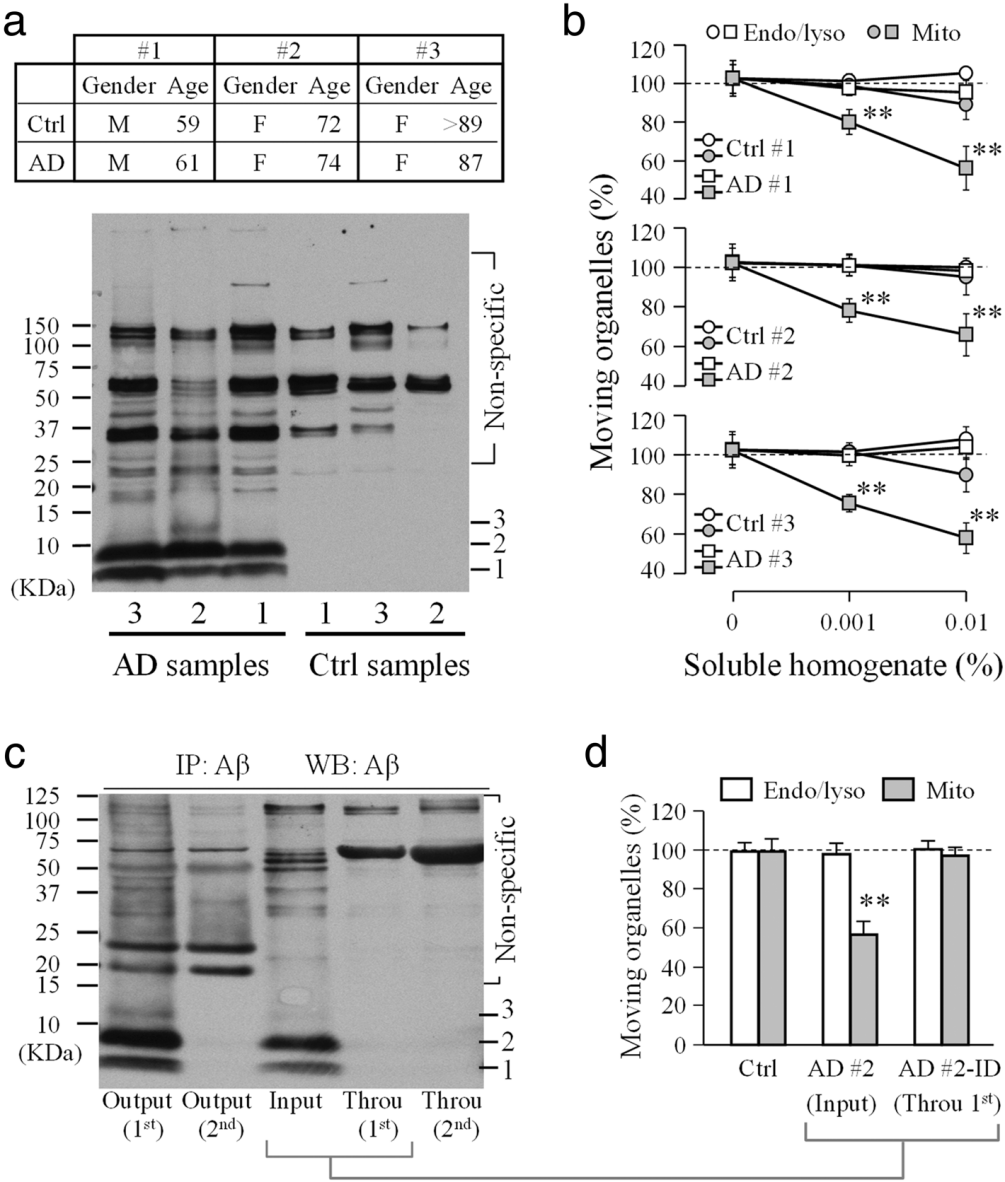
Selective inhibition of fast transport of mitochondria by Aβ oligomers from human brain tissues. **a** Western blots (lower) showing the presence of small Aβ oligomers in the soluble fraction of brain homogenates from the three age- and sex-matched AD patients and control patients as indicated in the table above. Aβ monomers (1) and dimmers (2) were clearly seen, whereas trimmers (3) were only seen in one AD sample. **b** Quantitative analysis showing the selective impairment of mitochondrial transport by different dilutions of soluble AD homogenates in KRB. **c** Western blots showing the effective depletion of Aβ molecules by immunoprecipitation (IP). Two runs of IP were performed and the supernatants (Throu 1^st^ and 2^nd^) and pellets (Output 1^st^ and 2^nd^) were blotted. **d** Quantitative analysis showing the effects of the soluble AD brain homogenate (AD#2, Input) and its supernatant after immunoprecipitation (Throu 1^st^) on mitochondrial transport. Error bars are SD. *p < 0.05 and ** *p* < 0.005 (Student’s *t-*test). Ctrl: control; AD, AD brain sample, Throu: supernatant through IP; Mito: mitochondria; Endo/lyso: late endosomes/lysosomes. Each condition was repeated at least three times from different rounds of cultures and was averaged from 10–20 cells

We next tested if Aβ oligomers from human brain tissues of Alzheimer’s patients exert similar acute and selective inhibition on mitochondrial transport. Soluble fractions of frontal cortex homogenates from three AD and three age- and gender-matched control brain samples were tested in this study. Western blotting confirmed the presence of Aβ oligomers in AD samples, of which dimers appear to be the predominant form in all three AD brain samples (Fig. 2a). Consistent with our results from synthetic Aβ-O, all three AD soluble homogenate, not that of control brain tissues, potently inhibited the transport of mitochondria without affecting the movement of endo/lysosomes (Fig. 2b). Similarly, the transport impairment of mitochondria was alleviated by LiCl, suggesting the involvement of GSK3β (Additional file 1: Figure S2). To determine if the effect of soluble AD homogenates on mitochondrial transport was indeed due to the Aβ molecules, we depleted Aβ molecules from the homogenates by immunoprecipitation using an Aβ antibody. As shown in the western blot (Fig. 2c), the beads effectively brought down Aβ monomers, small oligomers, and other aggregates after merely one round (see the Output 1st). The through solution (Throu 1st) contained essentially no Aβ molecules. When tested on hippocampal neurons, the through solution (Throu 1^st^) produced no effect on mitochondrial transport (Fig. 2d). Therefore, the inhibition of mitochondrial transport by AD soluble homogenates was caused by Aβ molecules. Together, these results indicate that acute impairment of mitochondrial transport may represent one of aberrant actions of Aβ oligomers on neurons, which could contribute to the disruption of neuronal functions.

**Fig. 3:**
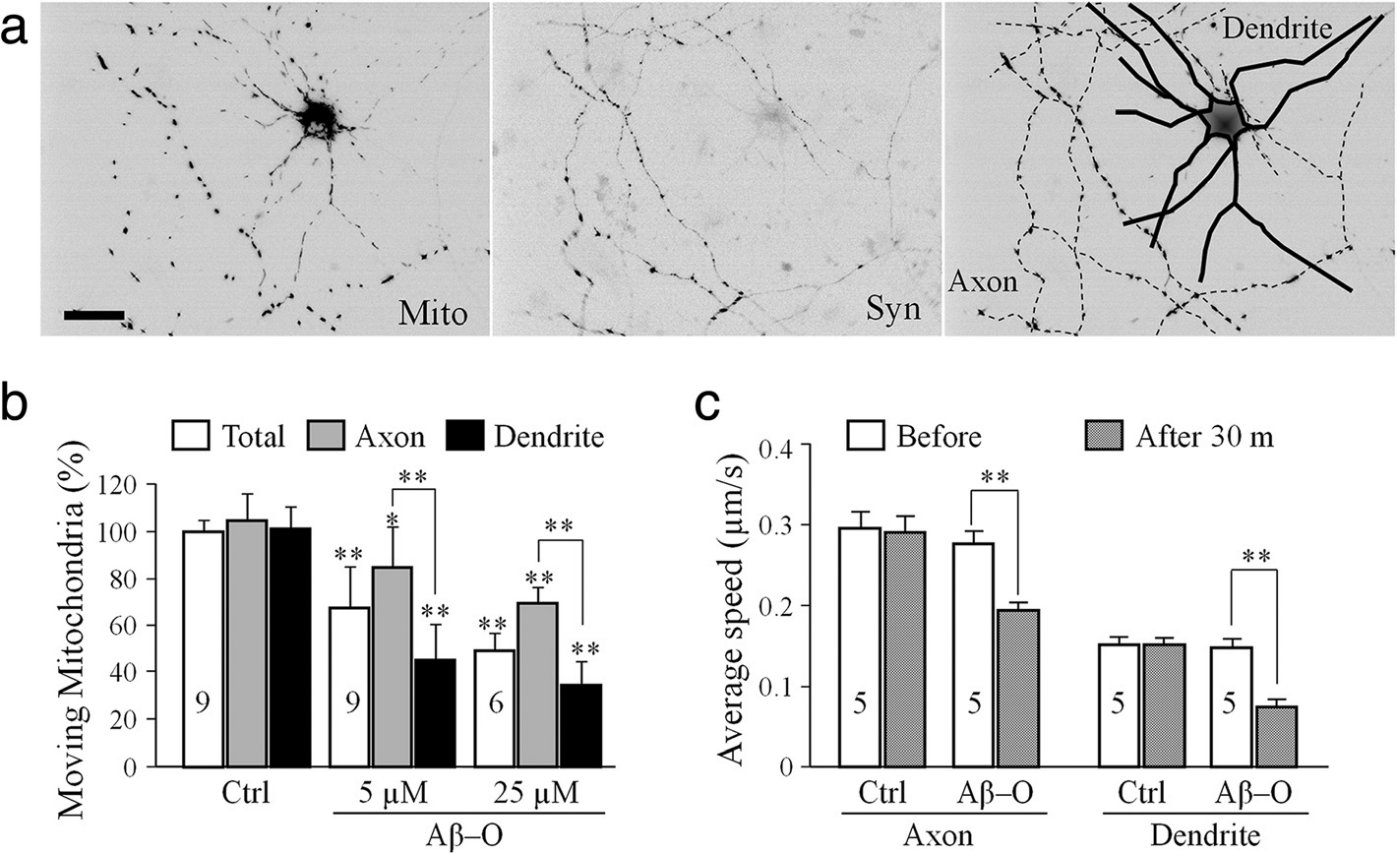
Acute impairment of mitochondrial transport in axons versus dendrites. **a** Representative images of a DIV6 hippocampal neuron expressing Mito-DsRed (Mito, left panel) and Synaptophysin-YFP (Syn, middle panel). Both images were used to identify the axonal and dendritic compartments of the neuron, as highlighted by the schematic drawing shown in the right panel. Here, solid lines represent the dendrites and dashed lines represent the axon. Scale bars: 20 μm. **b** and **c** Bar graphs showing the quantitative results on the acute impairment of mitochondrial transport in number (**b**) and in speed (**c**) by Aβ–O in axons and dendrites. Numbers indicate the number of neurons analyzed in each group (from at least three batches of culture). Error bars indicate SD (**b**) and SEM (**c**). *p < 0.05 and **p < 0.005 in comparison to the correspondent control or between axons and dendrites (Student’s *t-*test)

Neurons are polarized in their structure and function, as highlighted by their long axonal projection and elaborated dendritic branches. Mitochondria in axon and dendrite display different morphologies and patterns of movement which may due to their different functions. We next examined if Aβ impairment of mitochondrial transport is different for axonal and dendritic compartments. We expressed synaptophysin-YFP and mito-DsRed in hippocampal neurons for imaging. The axon and dendrite were distinguished by their morphology in combination with the accumulation of synaptophysin-YFP. Axons are much more elongated and thinner in comparison to the dendrites and highlighted by enriched synaptophysin-YFP signals when expressed at low levels (Fig. 3a). Similar time-lapse imaging was performed before and after Aβ-O application and the numbers of moving mitochondria in axonal and dendritic processes were quantified (Fig. 3b). We found that the Aβ-O caused more potent inhibition on mitochondrial transport in dendrites than that in axonal processes (*p* < 0.005, Student’s *t*-test; Fig. 3b). We also analyzed the movement speed of mitochondria and found that it was similarly reduced in axons and dendrites (Fig. 3c), although the reduction in dendrites appears to be larger than that in axons (~50 % vs. 30 %, *p* = 0.035 Student’s *t*-test). Given that the dendritic branches of a typical excitatory neuron receive and integrate a large number of synaptic inputs, Aβ impairment of mitochondrial trafficking in dendrites could play a crucial part in Aβ disruption of synaptic functions [19].

### Extended exposure to Aβ-O leads to mitochondrial fragmentation

**Fig. 4:**
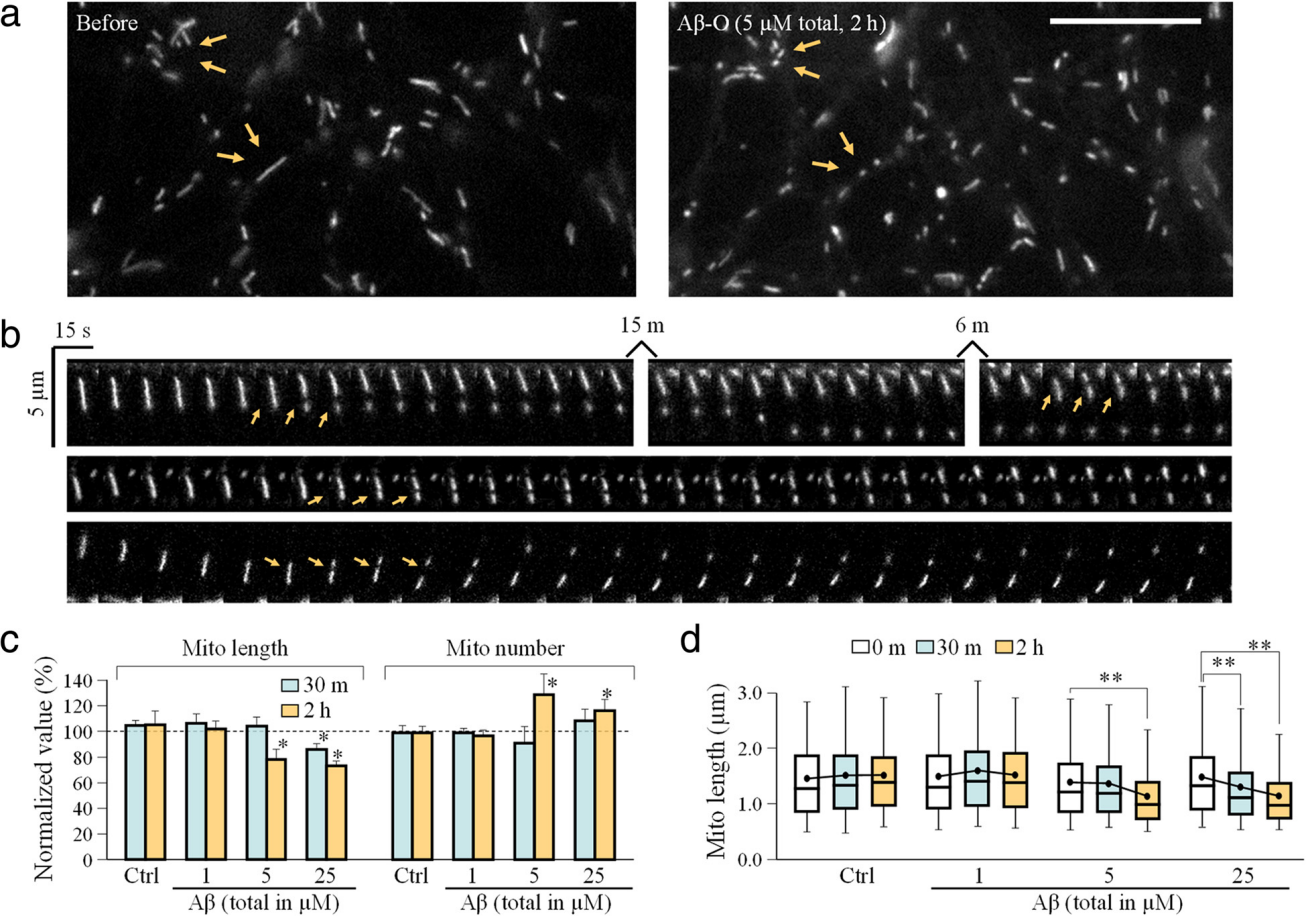
Mitochondrial fragmentation caused by prolonged exposure to high concentrations of Aβ oligomers. **a** Representative high-resolution images in upper panel showing mitochondria before (left panel) and after (right panel) 2 h exposure to 5 μM Aβ–O. Arrows indicate the location where fragmentation might have occurred after Aβ–O treatment. Scale bar: 10 μm. **b** Time-lapse sequences showing the fragmentation of individual mitochondria upon 25 μM Aβ exposure. Arrows indicate the site of fragmentation. **c** Quantitative analysis showing the reduction in mitochondrial length and the change in the total mitochondrial number in response to Aβ–O. Error bars indicate SD. **d** Box-whisker plots showing the changes in mitochondrial length after various exposures to different concentrations of Aβ–O. The boxes enclose the 25^th^ and 75^th^ percentiles; the middle lines mark the median, and the error bars denote the 5^th^ and 95^th^ percentiles. Error bars indicate SD. For (c–d), *p < 0.05 and **p < 0.005 when compared to the corresponding control (Student’s *t*-test). Each condition was repeated four times from different rounds of cultures and 600–1000 mitochondria were measured

While the rapid Aβ impairment of mitochondrial transport was not associated with any obvious changes in mitochondrial morphology, extended exposure to Aβ oligomers was found to cause mitochondrial fragmentation (Fig. 4). When the hippocampal neurons were exposed to 5 μM total Aβ (~500 nM oligomers) for 2 h, some long mitochondria were found to break into shorter pieces (arrows in the top panels of Fig. 4a). Time-lapse imaging sequences that followed individual mitochondria showed clearly the fragmentation process (Fig. 4b; 25 μM total Aβ). Since fragmentation results in shorter mitochondria, we assessed the fragmentation by measuring the length of hundreds of mitochondria. We found that mitochondrial fragmentation was only observed when the cells were exposed to 5 μM (total) Aβ-O for 2 h or 25 μM (total) Aβ-O for 30 min, as evidenced by the reduction in the average length of mitochondria (Fig. 4c). Consistently, Aβ-induced fragmentation increased the total number of mitochondria (Fig. 4c). Since not all the mitochondria exhibited fragmentation upon prolonged Aβ-O exposure, we presented the mitochondrial length of different groups in the box-whisker plot (Fig. 4d). These data show that Aβ oligomers caused mitochondrial fragmentation only at a higher dose than that causing transport impairment. Therefore, Aβ-induced mitochondrial fragmentation is unlikely the cause of transport impairment, but a separate effect induced by Aβ oligomers. Similar to Aβ impairment of mitochondrial transport, mitochondrial fragmentation induced by Aβ oligomers could also be abolished by inhibiting GSK3β with either LiCl or SB compounds (Additional file 1: Figure S3), supporting a central role for GSK3β in mediating Aβ effects.

### Distinct Aβ effects on mitochondria are mediated by specific pathways

We next investigated how Aβ oligomers elicit transport impairment and fragmentation of mitochondria, two effects with distinct time courses and dose-dependence. We first examined if prolonged Aβ-O exposure may disrupt the mitochondrial membrane potential leading to transport impairment and fragmentation. Live cell imaging using the TMRE dye [27, 28] showed that 2 h exposure to 5 μM (total concentration) Aβ-O did not affect the TMRE signal (Additional file 1: Figure S4). We next examined if Aβ oligomers caused cell death, which in turn resulted in mitochondrial transport defects and fragmentation. The cell viability assay [29] showed that cell death only started to be observed after 6 h exposure to Aβ-O (Additional file 1: Figure S5a). Similar results were also obtained using a different method in which a Hoechst dye was used to identify apoptotic nuclei (Additional file 1: Figure S5b). The very late onset of cell death upon Aβ-O exposure argues against cell death as the cause of the acute mitochondria defects induced by Aβ oligomers.

**Fig. 5:**
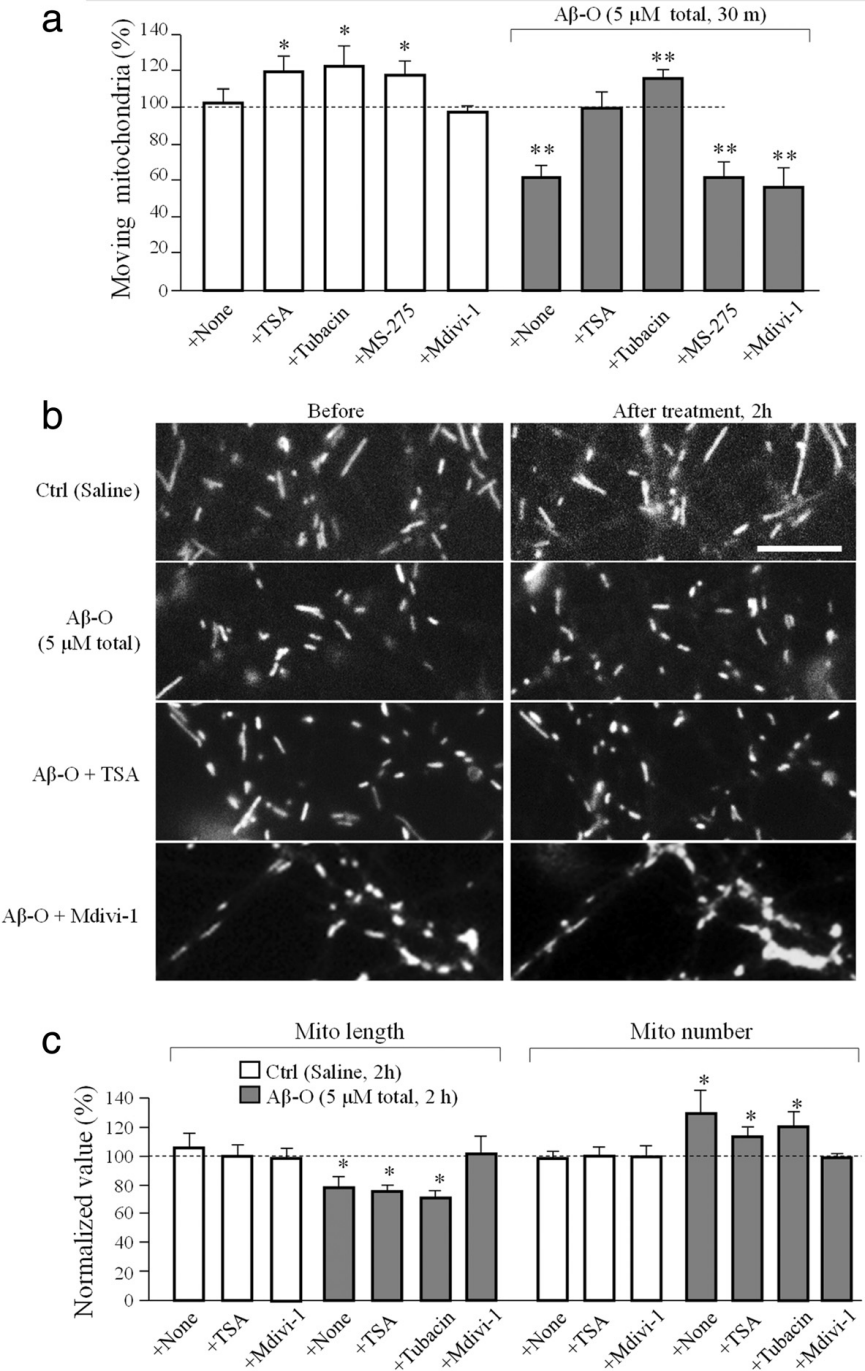
Effect of HDAC6 or Drp1 inhibition on Aβ–O induced mitochondria trafficking impairment and fragmentation. **a** Quantitative analysis showing the changes in moving mitochondria after 30 min exposure to different HDAC inhibitors (TSA 10 μM, tubacin 20 μM, MS-275 5 μM) or Drp1 inhibitor (Mdivi-1 20 μM) with and without Aβ–O. **b** Representative images showing the morphology of mitochondria before and after 2 h exposure to control or Aβ-O (5 μM total) without or with presence of TSA or Mdivi-1. Scale bar: 5 μm. **c** Bar graph showing the changes in the average mitochondrial length and total number in response to Aβ–O without and with HDAC6 or Drp1 inhibitions in bath. Numbers represent the number of dishes examined with a total of 10–20 cells for each data point. Error bars indicate SD. *p < 0.05 and **p < 0.005 in comparison to the corresponding control (Student’s *t* test)

Recent studies have implicated the cytoplasmic histone deacetylase 6 (HDAC6) in several neurodegenerative diseases including Alzheimer’s, Parkinson’s and Huntington’s diseases [30, 31]. Moreover, HDAC6 has been shown to regulate mitochondrial transport in hippocampal neurons [32]. We found that although acute exposure of hippocampal neurons to Aβ oligomers did not affect the expression of HDAC6 (Additional file 1: Figure S6 a and b), it did appear to increase the activities of HDACs in the cytosol and mitochondria as measured biochemically using the HDAC activity fluorometric assay kit (Additional file 1: Figure S6c). Since HDAC6 is the major HDAC in the cytoplasm, this result suggests that Aβ oligomers elevated the HDAC6 activities, which is supported by increased deacetylation of α-tubulin after Aβ-O exposure (Additional file 1: Figure S6 d–f). We thus tested if inhibition of HDAC6 could alleviate the Aβ effects on mitochondrial transport and structure. Application of trichostatin A (TSA), a selective inhibitor for the class I and II HDACs that includes HDAC6, completely abolished the acute impairment of mitochondrial transport by Aβ–O (Fig. 5a). We further tested tubacin, a specific HDAC6 inhibitor [33], and MS-275, a selective HDAC1 inhibitor [34] and found that only tubacin was able to abolish Aβ impairment of mitochondrial transport (Fig. 5a). Our kymograph analysis also showed that inhibition of HDAC6 by TSA or tubacin effectively arrogated Aβ–induced reduction in the transport speed of mitochondria (Additional file 1: Figure S7). Interestingly, neither TSA nor tubacin affected the mitochondrial fragmentation induced by Aβ–O (Fig. 5 b and c). Since mitochondria undergo fusion and fission, the latter is mediated by the dynamin-related GTPase (Drp1). We examined if mitochondria fission was involved in Aβ-induced fragmentation. We found that inhibition of Drp1 by the selective inhibitor mdivi*-*1 blocked Aβ-induced mitochondrial fragmentation (Fig. 5 b and c). However, mdivi-1 had no effect on the transport defects elicited by Aβ-O (Fig. 5a). Taken together, these results suggest that Aβ oligomers exert time-dependent and pathway-specific effects on mitochondrial transport and structure.

## Discussion

Stalled and deformed mitochondria in clusters are one of the outstanding signs of many neurodegenerative diseases [35, 36]. In AD, disruption in mitochondrial transport and function contributes to the disease pathogenesis and brain dysfunction [37–39]. While Aβ plaques are a hallmark of degenerating AD brains, soluble Aβ oligomers are believed to be the culprit underlying a variety of toxic effects on neuronal functions leading to the cognitive decline of the AD brain. While the adverse effects of Aβ oligomers on neurons and synapses are diverse and involve different targets, mitochondria are one of the major targets that Aβ oligomers negatively impact. In this study, we present evidence that Aβ oligomers elicit two acute effects on mitochondria: impairment of fast transport and fragmentation. Importantly we show that these two acute effects of Aβ oligomers involve different exposure times and doses of Aβ oligomer and are mediated by distinct signaling pathways. The selective impairment of mitochondrial transport is rapidly induced by low concentrations of by Aβ oligomers, whereas the fragmentation requires longer exposure and higher doses. While both Aβ effects on mitochondria can be blocked by inhibition of GSK3β, the transport impairment requires the activation of HDAC6 and fragmentation involves the GTPase drp1. Given that fast transport and fusion/fission of mitochondria are crucial for their function at subcellular locations, impairment of mitochondria transport and disruption of fusion/fission balance could argument the local function of mitochondria, such as at synapses.

Acute impairment of mitochondrial transport by Aβ molecules has been reported previously [22, 40]. This study has not only substantiated previous findings, but also provided additional insights. First, our findings show that Aβ oligomers selectively impair mitochondrial transport without affecting that of lysosomes and endosomes. Second, we present the evidence that Aβ oligomers from human AD brains exert the similar impairment of mitochondria transport to that of synthetic Aβ oligomers, indicating that this effect could potentially occur in human brains. Finally, our data suggest that Aβ oligomers negatively impact mitochondrial transport more effectively in dendrites than in axons. Mitochondria in axonal and dendritic compartments display different morphology and motility that may reflect their difference in functions in these two compartments [41]. The long and slender axons of neurons contain a limited space that is prone to road blocks and traffic jams for long range transport, especially under neurodegenerative conditions [17]. Dendrites, on the other hand, are shorter and thicker but form more elaborated arbors that function in receiving and integrating hundreds of synaptic inputs. Trafficking of synaptic receptors, mitochondria and other organelles, and mRNA-ribosomal complex occur actively in dendritic compartments and play a crucial role in postsynaptic functions [6, 10, 42, 43]. Our finding that mitochondrial transport in dendrites appears to be more severely inhibited by Aβ oligomers is consistent with the emerging view of Aβ adverse effects on postsynaptic structure and function [19, 44, 45]. Disrupted mitochondrial transport in dendrites could negatively impact the distribution and health of mitochondria to affect a number of mitochondria-dependent postsynaptic events, such as Ca^2+^ signaling, receptor trafficking, spine structure and plasticity, leading to synaptic dysfunction and degeneration [10].

The signaling mechanisms underlying the acute effects of Aβ oligomers on mitochondria remain to be elucidated. While inhibition of GSK3β appears to alleviate both Aβ effects, the transport impairment and fragmentation of mitochondria induced by Aβ oligomers are selectively abolished by the inhibition of HDAC6 and Drp1, respectively. HDAC6 is a member of class II HDACs that is localized mostly in the cytoplasm with a preference for non-histone proteins [46]. Increased HDAC6 level in cortex and hippocampus of AD brain has been reported [47]. HDAC6 has been found to deacetylate multiple non-histone proteins [48] such as α-tubulin [46, 49], tau [50], HSP90 [51] and cortactin [49]. The lysine 40 (K40) of α-tubulin is posttranslationally modified through acetylation in cells and HDAC6 is known to be the main deacetylase for this site, but this posttranslational modification does not appear to affect microtubule motor-based transport [52–54]. Therefore, other potential targets of HDAC6, such as tau [55], need to be investigated for their role in Aβ impairment of mitochondrial transport.

**Fig. 6:**
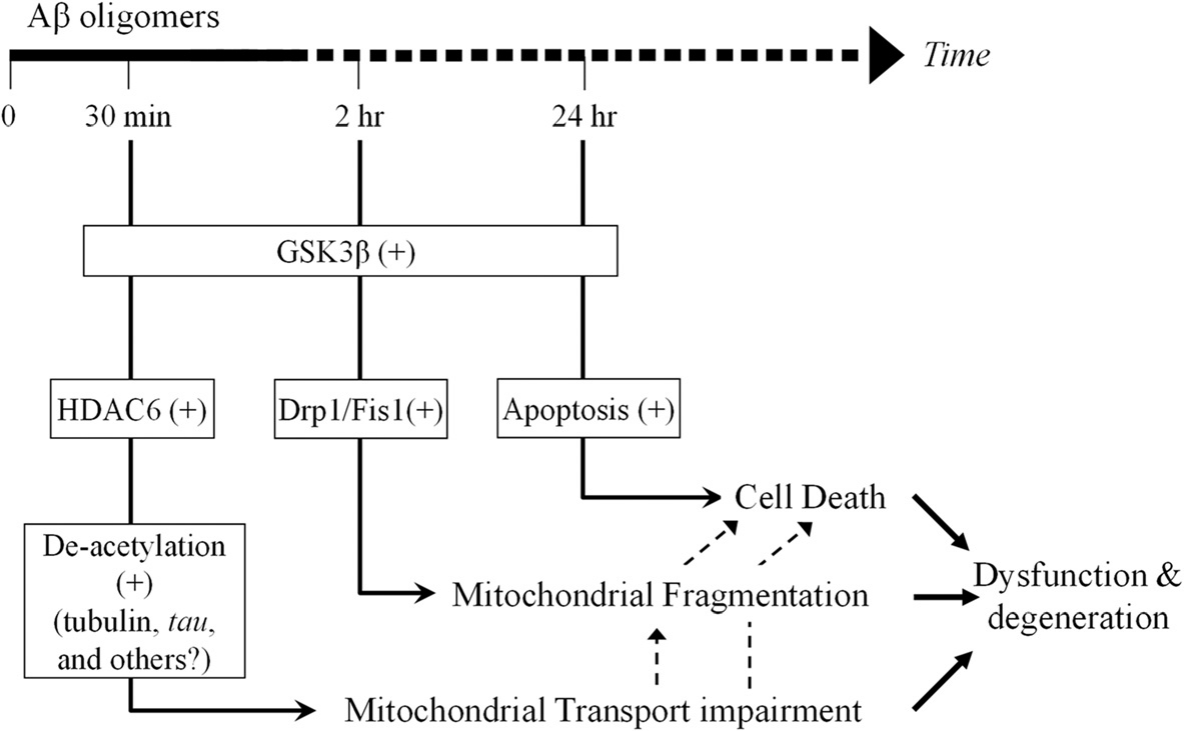
A schematic showing the temporal segregation and potential involvement of different pathways among three adverse effects of Aβ oligomers: mitochondrial transport impairment, fragmentation, and cell death. All three effects appear to involve GSK3β. Aβ impairment of mitochondrial transport involved HDAC6-mediated microtubule deacetylation. Mitochondrial fragmentation may involve the mitochondrial fission components of Drp1 and Fis1, whereas cell death is mediated by the apoptosis pathway. Dashed lines indicate potential alternative pathways

Our data suggest that the fragmentation induced by Aβ oligomers involves Drp1, a GTPase involved in mitochondria fission. Mitochondria are known to undergo fission and fusion, which are highly regulated to control the cellular distribution, health state, and function of mitochondria [56]. The Aβ targeting of Drp1 can result in excessive fission of mitochondria, leading to the disruption of mitochondrial distribution and function. Indeed altered expression levels of proteins involved in mitochondria fission and fusion have been observed in AD brains [57]. How Aβ oligomers activate Drp1 remains unknown at this moment. Drp1 can be regulated by a number of posttranslational modifications including phosphorylation [58], SUMOylation [59] and S-Nirosylation [60]. S-Nirosylation of Drp1 is increased in brain of human AD patients and inhibition of S-nitrosylation of Drp1 was shown to abrogate Aβ-triggered mitochondria fission, synaptic loss and neuronal damage [60]. On the other hand, GSK3β was found to interact with Drp1 to induce Drp1 phosphorylation, leading to mitochondria fragmentation in AD [61, 62]. Given that GSK3β has been shown to regulate HDAC6 activity [37], GSK3β could acts as a central node to transmit a wide range of Aβ actions, including HDAC6 activation and Drp1 phosphorylation to impact mitochondrial transport and mitochondrial fragmentation (Fig. 6). The next challenge is to elucidate how the signal divergence from GSK3β is achieved and regulated to generate distinct cellular effects underlying detrimental actions of Aβ oligomers.

## Additional file

Additional file 1: Supplemental figures S1-S7 and methods. (PDF 902 kb)

## Abbreviations

Aβ: Amyloid-β
Aβ-O: Aβ oligomers
CCD: Charge coupled device
GSK3β: Glycogen synthase kinase 3β
HDAC: Histone deacetylase
IP: Immunoprecipitation
NA: Numeric aperture
TSA: Trichostatin A
