# Supplementary material for: Amyloid β oligomers elicit mitochondrial transport defects and fragmentation in a time-dependent and pathway-specific manner
Source: Mol Brain. 2016 Aug 17;9:79. doi: 10.1186/s13041-016-0261-z (PMC4989350; doi:10.1186/s13041-016-0261-z)
Supplement: Additional file 1: — Supplemental figures S1-S7 and methods. (PDF 902 kb) [file 13041_2016_261_MOESM1_ESM.pdf]

Additional file 1

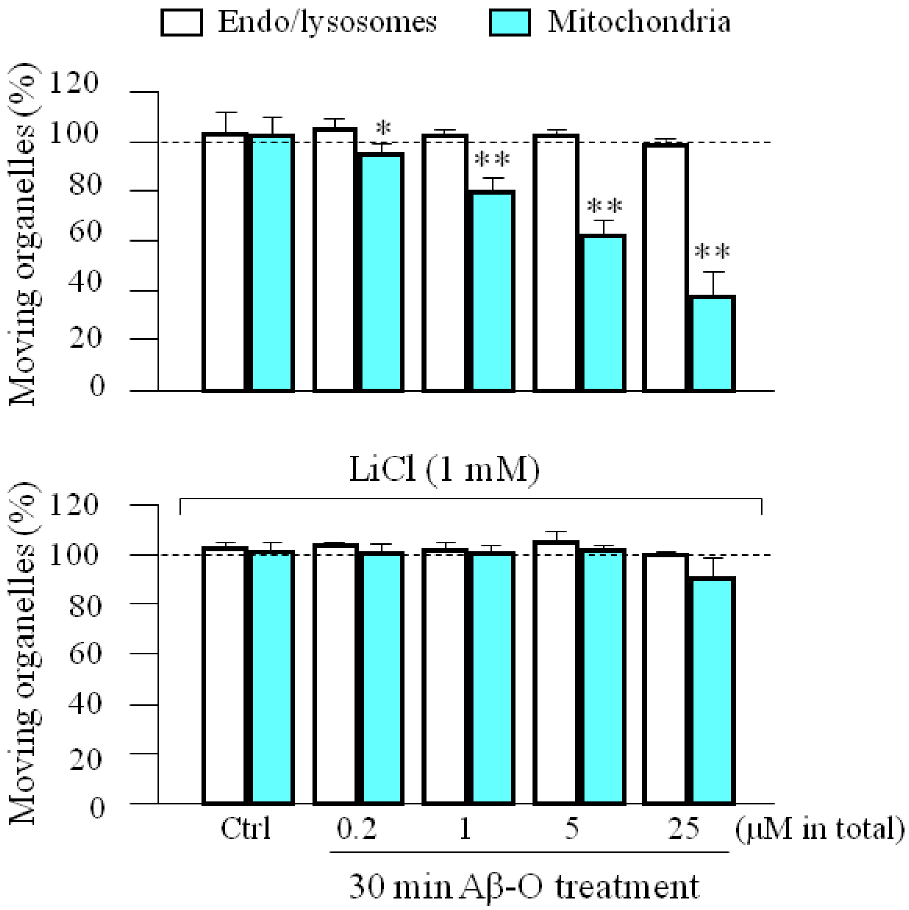

Figure S1. Abolishment of Aβ effects on mitochondrial transport by LiCl. The bar graph shows the percentage of moving mitochondria before and after 30min exposure to different concentrations of Aβ–O, without (upper) and with (lower) LiCl (1 mM) in bath. Each condition was repeated at least three times from different rounds of cultures and was averaged from 10 -20 cells. Error bars indicate the standard deviation (SD). *p<0.05 and ** p<0.005 in comparison to the corresponding control (Student's t-test).

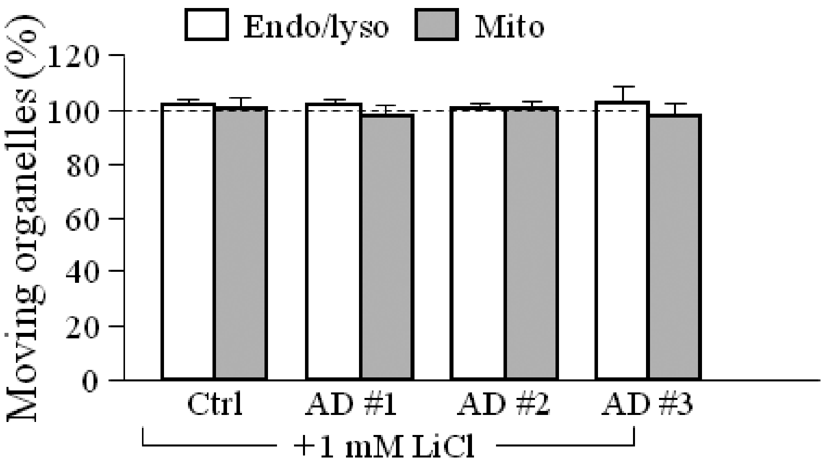

Figure S2. Abolishment of soluble AD brain samples effect on mitochondrial transport by LiCl. Quantitative analysis showing that inhibition of GSK3β by LiCl blocked the transport impairment of mitochondria by soluble AD brain samples. Each condition was repeated at least three times from different rounds of cultures and was averaged from 10 -20 cells. Error bars indicate the standard deviation (SD). *p<0.05 and ** p<0.005 in comparison to the corresponding control (Student's t-test).

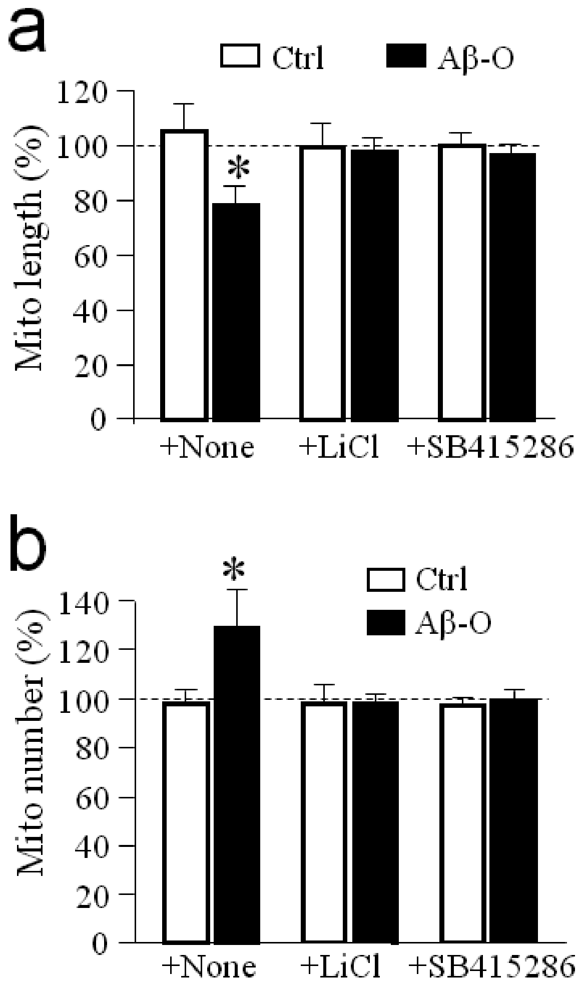

Figure S3. Inhibition of GSK3β preventing Aβ-induced mitochondrial fragmentation. Quantitative analysis showing the changes in average mitochondrial length (a) and total mitochondrial number (b) in response to Aβ–O exposure (5 μM, 2 h) without and with different GSK3β inhibitors in bath. Lithium chloride (LiCl, 1 mM) and SB415286 (10 μM) were added to the cells 20 min before Aβ–O exposure. Error bars indicate SD. *$p<0.05$ in comparison to the corresponding control. Around 1000 cells from three repeats were measured.

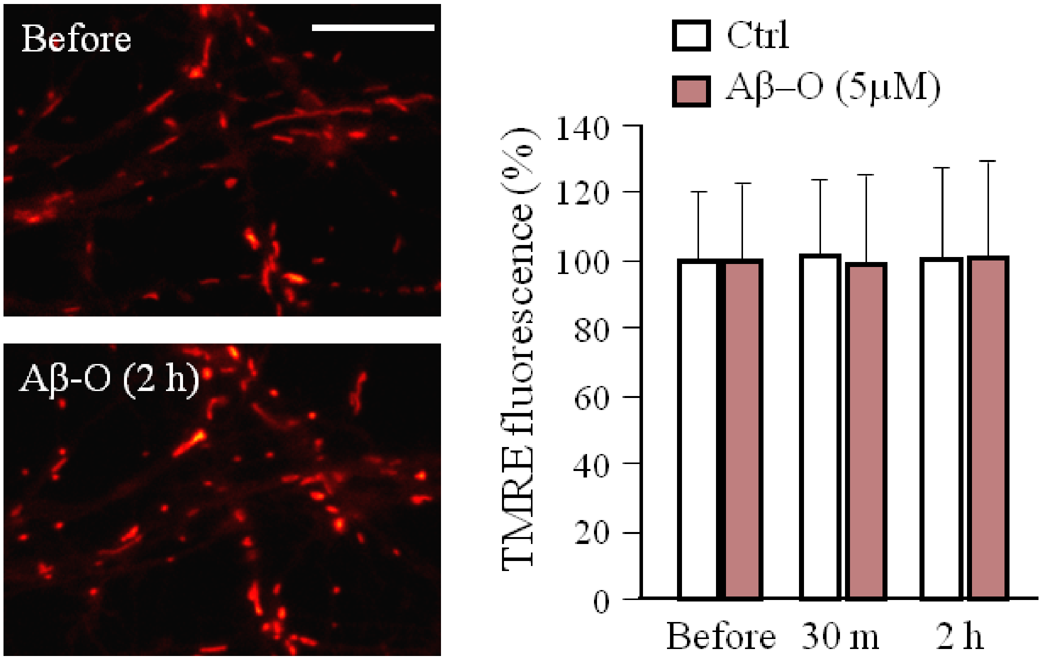

Figure S4. Effect of Aβ-O on mitochondria membrane potentials. TMRE imaging showing no effect Aβ-O on mitochondrial potential. Representative images are shown on the left and normalized TMRE intensities of mitochondria (600 – 1000 mitochondria in each group) are shown in the bar graph on the right. Scale bar: 10 μm; error bars: SD.

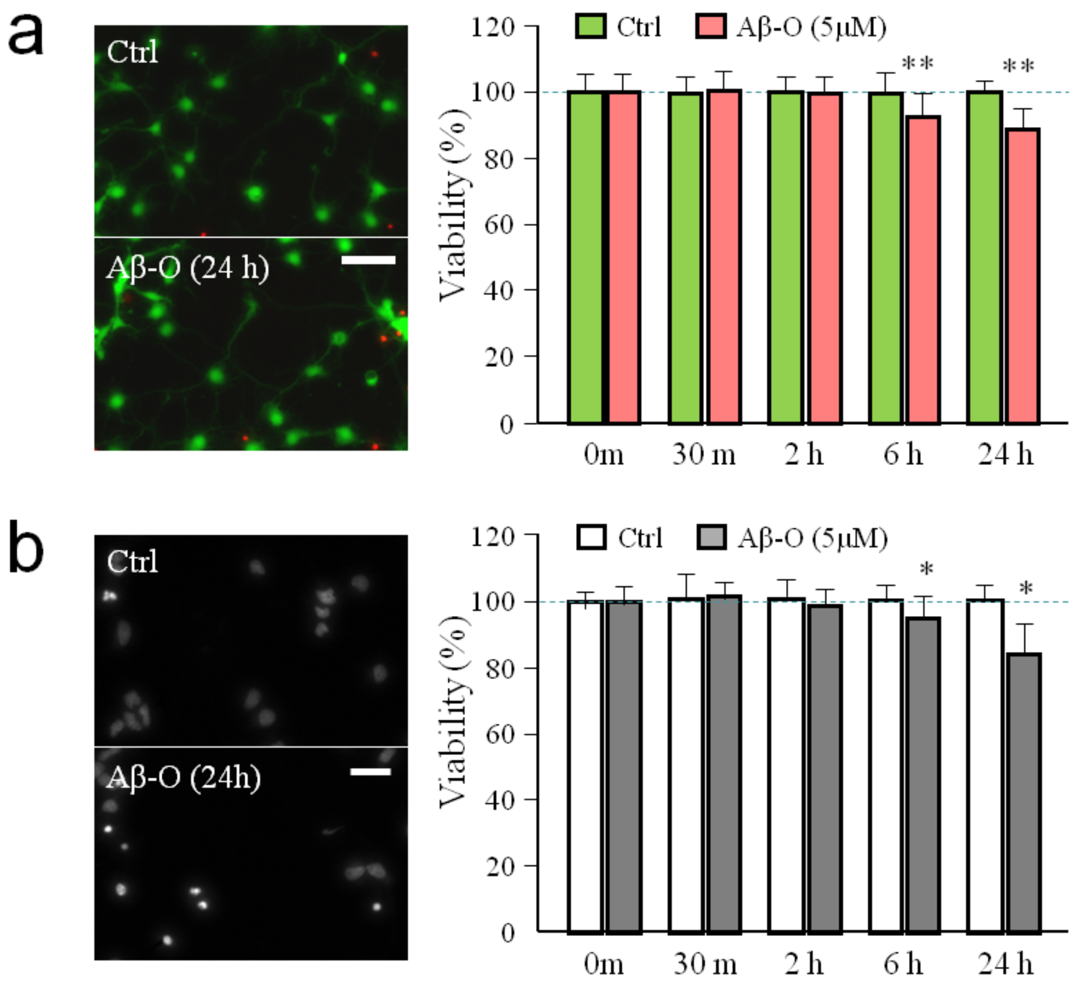

Figure S5. Effect of Aβ-O on cell death. (***a***) Results from the viability assay using the Live/Dead kit. Representative fluorescent images of hippocampal neurons exposed to the control and Aβ oligomers. The live cells were labeled by calcein AM (green), and the dead cells were labeled by EthD-1 (red). Quantified percentages of live cells are shown in the bar graph. Scale bar: 50µm. p<0.05 and **p<0.005 in comparison to the corresponding control (Student's *t*-test). Each condition was repeated at least three times from different rounds of cultures and was averaged from around 1000 cells. (***b***) Assessment of cell death induced by Aβ oligomers using Hoechst staining. The fluorescent images show examples of Hoechst staining of hippocampal neurons without and with 24 hr exposure to Aβ–O (5 µM). Scale bar: 50µm. The bar graph shows the normalized cell viability at different times after exposure to Aβ–O. Error bars represent S.D.. Each group includes results from over 1,000 cells from three different batches of hippocampal cultures.

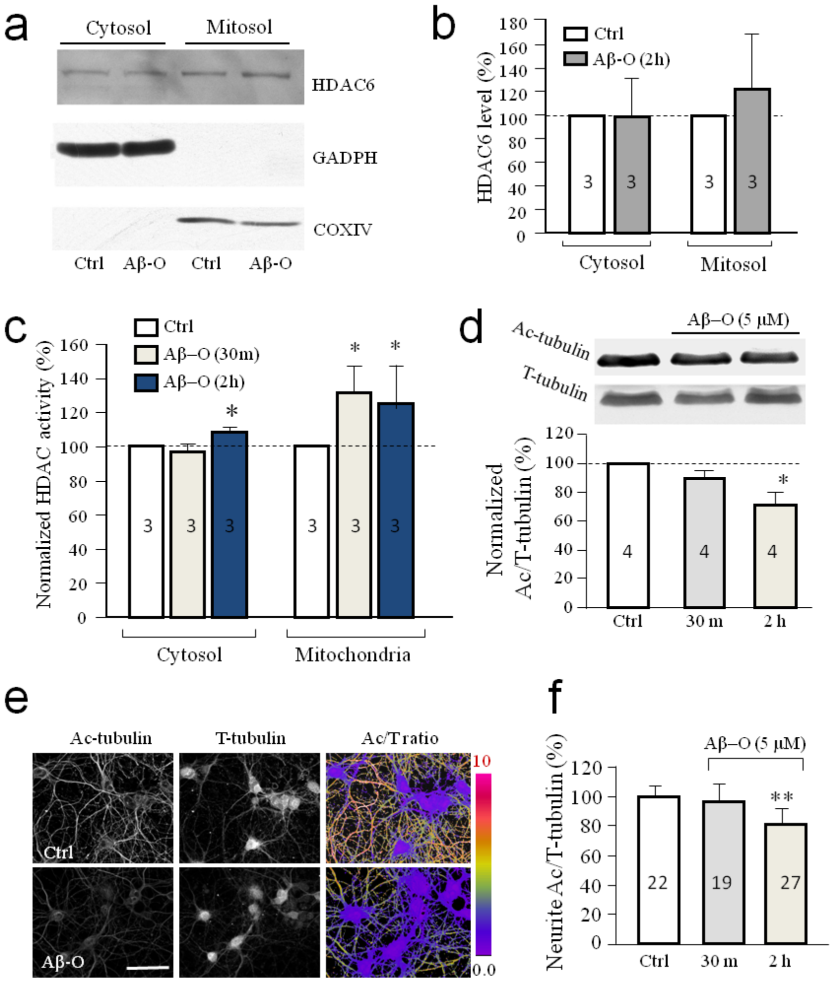

Figure S6. Increase in HDAC activity in hippocampal neurons exposed to Aβ oligomers. (***a***) Western blots of HDAC6 in cytosolic and mitochondrial fractions from cultured hippocampal neurons showing little changes in HDAC protein expression level after exposure to Aβ oligomers (5 µM total, 2h). GADPH: Glyceraldehyde-3-Phosphate Dehydrogenase. COXIV: Cytochrome C oxidase 4. The quantification results of the blots are shown in (***b***). Numbers represent the number of repeated WB. (***c***) Measurements of HDAC activities in separated cytosolic and mitochondrial fractions showing increased total HDAC activities in hippocampal neurons after exposure to Aβ oligomers. Numbers represent the number of repeated activity tests. (***d***) Western blots showing a reduction in acetylated α-tubulin (Ac-tubulin), not total tubulin (T-tubulin), after Aβ-O exposure (5 µM, 2 h). Quantifications are shown in the bar graph below. Numbers represent the number of repeated WB. (***e***) Immunofluorescent images of Ac-tubulin (left panel), T-tubulin (middle panel), and the AC/T-tubulin ratio (right panel) in control (Ctrl, upper panels) and Aβ-treated (lower panels) neurons. Scale bar: 50 µm. (***f***) Bar graph showing the quantifications of AC/T-tubulin ratio for different groups of neurons. Numbers represent the number of neurons examined. Error bars indicate SD. **p*<0.05 and ***p*<0.005 in comparison to the corresponding control (Student's *t* test).

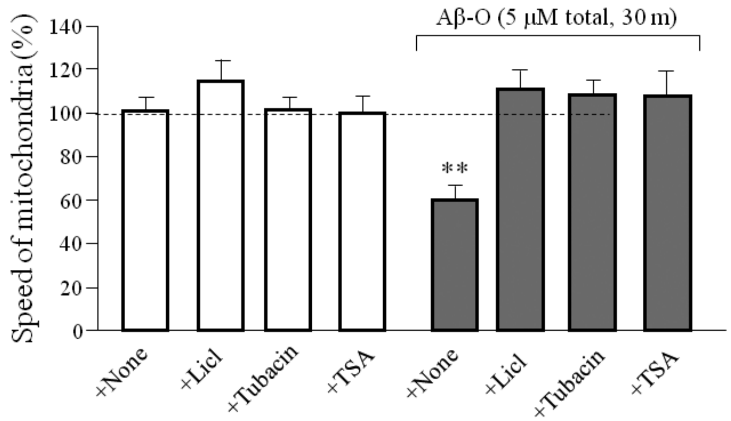

Figure S7. Inhibition of GSK3β and HDAC prevents the speed reduction of mitochondria transport induced by Aβ. Quantitative analysis showing the changes in average moving mitochondria speeds after 30min exposure to different GSK3β and HDAC inhibitors with and without Aβ–O. Here, the speed was normalized against the average speed in the control 5 min period (before treatment). Error bars indicate SD. $^{**}p<0.005$ in comparison to the corresponding control (Student's *t* test).

Supplementary Materials and Methods

Measurement of mitochondrial membrane potential and cell viability

The fluorescent dye, TMRE, was used to examine the mitochondrial membrane potential and its change [1, 2]. Hippocampal neurons were incubated with 100 nM of TMRE in KRB for 20 min at 37 °C in a 5% CO_2 incubator and live-cell imaging was performed in KRB buffer containing 100 nM TMRE for equilibrium. All solutions for exchange contained the same concentration of TMRE (100 nM) as the original equilibration buffer. The fluorescent images were acquired using the same optical settings. The fluorescence intensity of each mitochondrion was measured using ImageJ and normalized to the average intensity of the same region before treatment. Normalized data are shown as percentage changes from the average intensity of the dye measured before each treatment. Approximately 1000 mitochondria from four dishes were counted for each group.

To assess the numbers of live and dead cells, we used the LIVE/DEAD Viability/Cytotoxicity kit (Invitrogen) that contains calcein AM and ethidium homodimer-1 (EthD-1) [3]. Briefly, hippocampal cells were incubated with 1 µm calcein AM and 2 µm EthD-1 for 30 min at 37°C, washed with KRB, and imaged by fluorescence microscopy. Ten randomly selected fields near the center of each dish were imaged, and the numbers of live (calcein labeled, green fluorescence) and dead (EthD-1 labeled, red fluorescence) cells were counted. Approximately 1,000 cells from three independent experiments were examined for each condition. Alternatively, we used the fluorescent DNA dye Hoechst 33342 (Sigma) to quantify apoptotic cell death [4]. Here, hippocampal neurons with and without Aβ treatments were fixed with 4% paraformaldehyde (30 min) and incubated with 10 µg/ml Hoechst 33342 for 30 min. Apoptotic cells with fragmented or condensed nuclei were identified by fluorescent microscopy. The

percentage of live cells (of the total cells) was calculated and used for assessing Aβ effects on cell apoptosis. Approximately 1000 cells were counted for each group and repeated 2-3 times.

<u>HDAC activity assay</u>

We used the HDAC activity fluorometric assay kit (Enzo Life Science, Farmingdale, NY) to measure the cytosolic deacetylase activity before and after Aβ exposure according to the manufacturer's instructions. Briefly, hippocampal neurons were scrapped off the culture dishes and washed twice with PBS and homogenized in lysis buffer (10 mM Tris, pH 7.5, 10 mM NaCl, 3 mM $MgCl_2$, 0.05% Nonidet P-40 and 1 mM EGTA, supplement with protein inhibitor cocktail). The homogenates were centrifuged at 3000 rpm to remove nuclei and any other insoluble fractions. The soluble homogenates were directly used for HDAC activity assay or subjected to separation of mitochondrial and cytosolic factions using a mitochondria extraction kit (Active motif, Carlsbad, CA). Each sample (15 µl) was added to a 96-well plate in 10µl HDAC assay buffer (Enzo Life Science, Farmingdale, NY). A fluorometric acetylated substrate was added and the reaction was allowed for 1 h at room temperature, followed by incubation with a developer for 10-15min. Enzymatic activity was evaluated in a FLX-800 fluorescence microplate reader (excitation: 485 nm, emission: 530nm). HeLa nuclear extract provided in the kit was used as positive control.

Reference cited:

1. Nicholls DG, Budd SL. Mitochondria and neuronal survival. Physiological reviews. 2000;80(1):315-60. Epub 2000/01/05. PubMed PMID: 10617771.
2. Loew LM, Tuft RA, Carrington W, Fay FS. Imaging in five dimensions: time-dependent membrane potentials in individual mitochondria. Biophysical journal. 1993;65(6):2396-407. Epub 1993/12/01. doi: 10.1016/S0006-3495(93)81318-3. PubMed PMID: 8312478; PubMed Central PMCID: PMC1225980.

3. Vaughan PJ, Pike CJ, Cotman CW, Cunningham DD. Thrombin receptor activation protects neurons and astrocytes from cell death produced by environmental insults. The Journal of neuroscience : the official journal of the Society for Neuroscience. 1995;15(7 Pt 2):5389-401. Epub 1995/07/01. PubMed PMID: 7623161.
4. Jordan J, Galindo MF, Miller RJ. Role of calpain- and interleukin-1 beta converting enzyme-like proteases in the beta-amyloid-induced death of rat hippocampal neurons in culture. Journal of neurochemistry. 1997;68(4):1612-21. PubMed PMID: 9084433.
